# iTRAQ-based quantitative proteomic analysis reveals the lateral meristem developmental mechanism for branched spike development in tetraploid wheat (*Triticum turgidum* L.)

**DOI:** 10.1186/s12864-018-4607-z

**Published:** 2018-04-02

**Authors:** Shulin Chen, Juan Chen, Fu Hou, Yigao Feng, Ruiqi Zhang

**Affiliations:** 1 College of Agronomy, National Key Laboratory of Crop Genetics and Germplasm Enhancement/JCIC-MCP, Nanjing Agricultural University, Nanjing, 210095 China; 2 College of Agronomy, Henan Agricultural University/Collaborative Innovation Center of Henan Grain Crops, Zhengzhou, 450002 China

**Keywords:** Branched spike, ITRAQ, Quantitative proteomics, Spikelete, *Triticum turgidum* L

## Abstract

**Background:**

Spike architecture mutants in tetraploid wheat (*Triticum turgidum* L., 2n = 28, AABB) have a distinct morphology, with parts of the rachis node producing lateral meristems that develop into ramified spikelete (RSs) or four-rowed spikelete (FRSs). The genetic basis of RSs and FRSs has been analyzed, but little is known about the underlying developmental mechanisms of the lateral meristem. We used isobaric tags for relative and absolute quantitation (iTRAQ) to perform a quantitative proteomic analysis of immature spikes harvested from tetraploid near-isogenic lines of wheat with normal spikelete (NSs), FRSs, and RSs and investigated the molecular mechanisms of lateral meristem differentiation and development. This work provides valuable insight into the underlying functions of the lateral meristem and how it can produce differences in the branching of tetraploid wheat spikes.

**Results:**

Using an iTRAQ-based shotgun quantitation approach, 104 differential abundance proteins (DAPs) with < 1% false discovery rate (FDR) and a 1.5-fold change (> 1.50 or < 0.67) were identified by comparing FRS with NS and RS with NS genotypes. To determine the functions of the proteins, 38 co-expressed DAPs from the two groups were annotated using the Gene Ontology and Kyoto Encyclopedia of Genes and Genomes analytical tools. We discovered that proteins involved in “post-embryonic development” and “metabolic pathways” such as carbohydrate and nitrogen metabolism could be used to construct a developmentally associated network. Additionally, 6 out of 38 DAPs in the network were analyzed using quantitative real-time polymerase chain reaction, and the correlation coefficient between proteomics and qRT-PCR was 0.7005. These key genes and proteins were closely scrutinized and discussed.

**Conclusions:**

Here, we predicted that DAPs involved in “post-embryonic development” and “metabolic pathways” may be responsible for the spikelete architecture changes in FRS and RS. Furthermore, we discussed the potential function of several vital DAPs from GO and KEGG analyses that were closely related to histone modification, ubiquitin-mediated protein degradation, transcription factors, carbohydrate and nitrogen metabolism and heat shock proteins (HSPs). This work provides valuable insight into the underlying functions of the lateral meristem in the branching of tetraploid wheat spikes.

**Electronic supplementary material:**

The online version of this article (10.1186/s12864-018-4607-z) contains supplementary material, which is available to authorized users.

## Background

In cereal crops, grain yield depends mainly on spikes per unit crop area, grains per spike, and grain weight. Of these factors, grain number per spike is influenced by inflorescence architecture and floral development processes [1]. Therefore, understanding the mechanisms underlying inflorescence architecture is crucial for both developmental and agronomic yield-related reasons. The spike architecture of wheat, also known as the compound inflorescence, is normally composed of sessile spikelets arranged in two opposite rows along the main axis (the rachis), with each spikelet producing 3–5 florets. Mutations of spike architecture in tetraploid wheat (*Triticum turgidum* L., 2n = 28, AABB) can produce branched spikes or supernumerary spikelets. These include ramified spikelet (RS) and four-rowed spikelet (FRS) mutants, which have more spikelets and kernels per spike and may increase the yield potential of wheat [2–4]. RS mutants have additional spikelets on an extended rachilla that develop from the axillary region, whereas FRS mutants have lateral sessile spikelets that develop from the axillary region forming two spikelets per rachis node [2, 3]. Although the RS and FRS phenotypes are different from the supernumerary spikelet (SS) phenotype in tetraploid wheat, the lateral meristem primordium still appears between the glume and lemma [2]. Genetic studies have demonstrated that the *TtBH/WFZP* gene determines the formation of the lateral meristem in wheat. This gene is orthologous to the AP2/EREBP-like transcription factor genes branched silkless1 (*BD1*) in maize and frizzy panicle (*FZP*) in rice [5–8]. Two *TtBH/WFZP* homoeologous genes are located on the 2AS and 2BS chromosomes of tetraploid wheat, but only mutations in *TtBH/WFZP-A* affect the development of the lateral meristem [2]. The *EREBP*/*APETELA2* (*AP2)-*type family genes are important for determining the degree of ramification in lateral meristems and regulating the spatiotemporal expression of spikelet meristem genes in *Triticeae* cereals [8]. These genes encode proteins with one or two AP2 domains that can bind to promoter GCC boxes and regulate the expression levels of genes important in abiotic and biotic stress resistance [9, 10]. Despite the successful isolation and characterization of several AP2-family genes that function in spike development, the molecular networks that govern lateral meristem formation for spike branches in *Triticeae* cereals remain unclear.

Proteomic approaches have provided insight into the mechanism of complex biological processes at the protein level and have been used widely to study *Triticeae* cereals. These methods also offer the possibility of simultaneously studying the chromosome locations of genes, protein-protein interaction networks, enzyme complexes, and post-translational modifications that are important for understanding plant development [11]. A particularly powerful proteomic analysis technique is the isobaric tags for relative and absolute quantitation (iTRAQ) method, which can identify numerous proteins and provide more reliable quantitative information than conventional analysis by two-dimensional gel electrophoresis [12, 13]. The iTRAQ method has been used to analyze the quantitative proteomics of wheat in response to powdery mildew [14], stripe rust [15], and drought [16], and during grain development [17]. However, quantitative proteomic research has not been used to examine how the lateral meristem produces branched spikes in wheat.

To investigate how the lateral meristem forms branched spikes at the proteomic level, we compared the immature spike proteome of three tetraploid wheat near isogenic lines (NILs) with normal spikelets (NSs), FRSs, and RSs using the iTRAQ technique. The proteomic data were subsequently integrated into a network containing several regulatory pathways to generate a systematic representation of the processes occurring in the lateral meristem of tetraploid wheat. These data will provide a foundation for understanding the regulation of the lateral meristem in branched wheat spike formation.

## Methods

### Plant materials

**Fig. 1:**
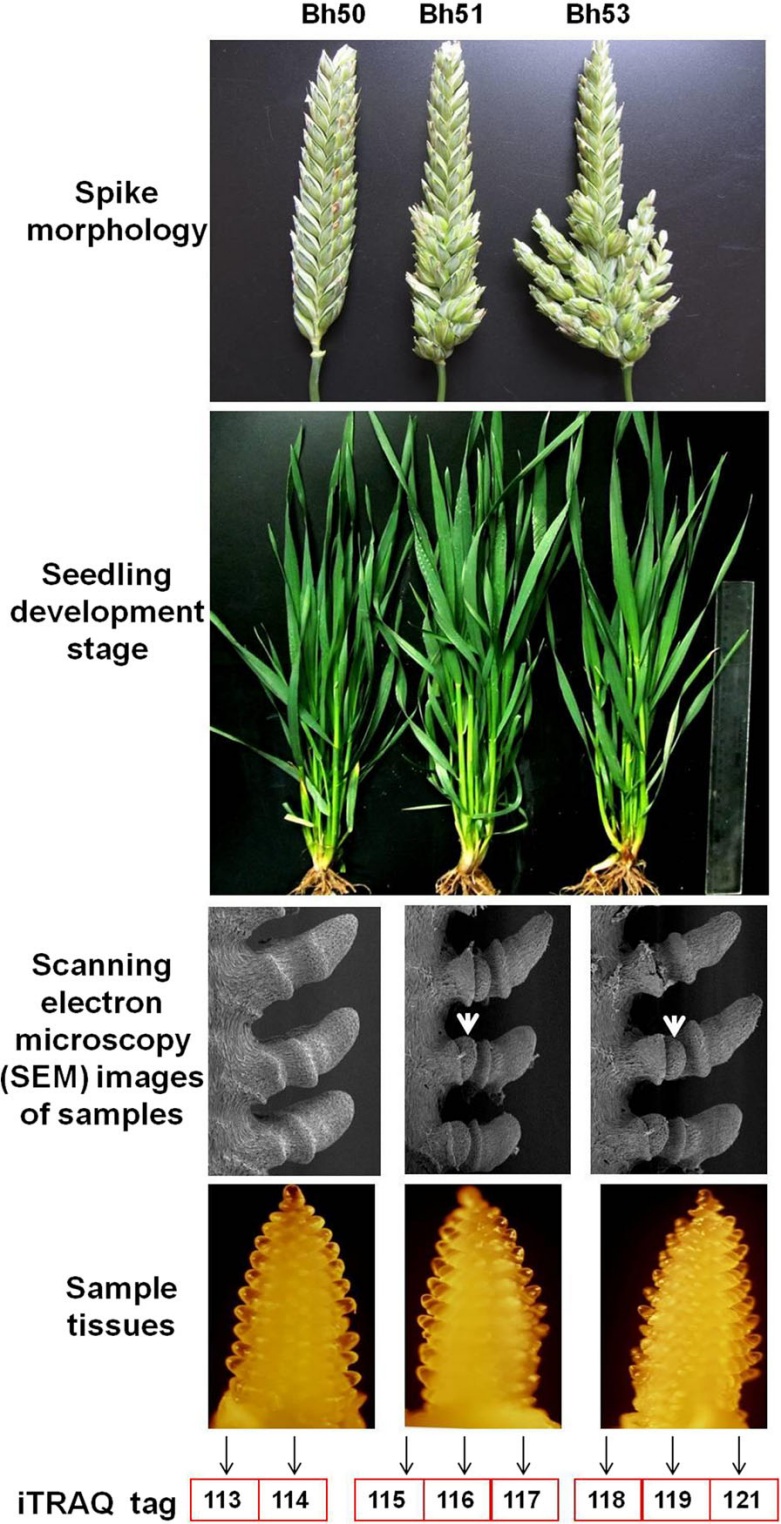
Morphological characteristics of normal spikelet (NS), four-rowed spikelet (FRS), and ramified spikelet (RS) near isogenic lines (NILs) of tetraploid wheat

Three tetraploid wheat (*Triticum turgidum* L.) NILs with different spike phenotypes, including bh50 with NSs, bh51 with FRSs, and bh53 with RSs, were used for proteomic analysis (Fig. 1). The NILs were planted in a greenhouse, and immature spike tissue was harvested from the main tiller of each plant at the spikelet initiation stage (following the double ridge stage, Fig. 1).

### Protein extraction

Total protein from immature spikelet tissue was extracted when the spikelets were approximately 3 mm long using a Plant Total Protein Extraction Kit (Sigma-Aldrich, St. Louis, MO, USA), in accordance with the manufacturer’s instructions. In total, 250 mg of plant tissue was ground in liquid nitrogen and extracted using phenol, in accordance with the method described by Isaacson et al. (2006) with minor modifications [18]. The mixture was purified using acetone, and the purified proteins were dissolved in lysis solution at 30 °C for 1 h. After centrifugation, the concentration of extracted protein was determined using the Bradford method (Bio-Rad, Hercules, CA, USA, and proteins were stored at − 80 °C for iTRAQ analysis [19].

### Protein quantitation and iTRAQ labeling

Samples containing approximately 10 μg of protein were run on 12% SDS-PAGE gels and were visualized using Coomassie brilliant blue stain, in accordance with Candiano’s protocol [20]. The stained gels were scanned using an Image Scanner (GE Healthcare, Chicago, IL, USA) at a resolution of 300 dots per inch. Aliquots containing 100 μg were obtained from each sample by adding 5 volumes of cold acetone. A total of 50 μL of dissolution buffer (4% SDS, 100 mM DTT, 150 mM Tris-HCl, pH 8.0) and 4 μL of a solution containing reducing agents (8 M urea, 150 mM Tris-HCl, pH 8.0) were added, and the mixture was incubated at 60 °C for 1 h. Subsequently, 2 μL of cysteine-blocking reagents were added at room temperature to collect the peptide. The resulting peptide mixture was labeled using the 8-plex iTRAQ reagent, in accordance with the manufacturer’s instructions (Applied Biosystems, Inc., Foster City, CA, US). The bh50 samples were labeled 113 and 114; bh51 samples were labeled 116, 117, and 118; and bh53 samples were labeled 118, 119, and 121 (Fig. 1). All labeled samples were then multiplexed and vacuum-dried for further identification.

### 2D-LC separation and RPLC-MS/MS analysis

Prior to LC-MS/MS analysis, excess labeling reagent was removed by strong cation exchange chromatography. Peptides were separated using an Agilent 1200 HPLC System (Poly-SEA 5 μL 300 Å 2.0 × 150 mm with 215 nm and 280 nm UV detection; Agilent, Santa Clara, CA, USA) at 0.3 ml/min using a nonlinear binary gradient starting with buffer A and transitioning to buffer B (10 mM KH_2_PO_4_, pH 3.0, 500 mM KCl, 25% acetonitrile). The first fraction was collected at 0–5 min. Thereafter, fractions were collected at 4 min intervals from 6 to 44 min, and the final fraction was collected at 45–50 min to generate a total of 12 fractions. Each fraction was dried in a vacuum freeze dryer for LC-MS/MS analysis. Peptide samples were resuspended in Nano-RPLC buffer A (0.1% formic acid, 2% acetonitrile). Online Nano-RPLC was performed using an Eksigent nanoLC-Ultra 2D system (AB SCIEX, Framingham, MA, USA). Proteins were identified using two technical replicates for each biological replicate. Samples were loaded onto a C_18_ nanoLC trap column (100 × 3 cm, C_18_, 150 Å) and washed with buffer A at 2 μL/min for 10 min. An elution gradient of 5%–3.5% acetonitrile (0.1% formic acid) over 70 min was used on an analytical ChromXP C_18_ column (75 μm × 15 cm, C_18_, 3 μm 120 Å) with a spray tip. Data were acquired using a Triple TOF 5600 system (AB SCIEX) fitted with a Nanospray III source and a pulled quartz tip as the emitter. For information-dependent acquisition, survey scans were acquired over 250 ms, and as many as 35 product ion scans were collected if these exceeded a threshold of 150 counts per second with a charge of 2^+^ to 5^+^.

### iTRAQ data analysis

MS/MS spectra were processed with Protein Pilot software (Protein Pilot 4.0; AB SCIEX) against the *Triticum aestivum* database using the Paragon algorithm [21]. An automatic decoy database search strategy was employed to estimate the false discovery rate (FDR) using the PSPEP (Proteomics System Performance Evaluation Pipeline Software, integrated in the Protein Pilot software), which was calculated as the false positive matches divided by the total matches. Proteins were identified using the following parameters: sample type = iTRAQ 8-plex (peptide-labeled), Cys; alkylation = iodoacetamide; digestion = trypsin; instrument = TripleTOF5600 (AB SCIEX); database = *Triticum aestivum*.fasta. The iTRAQ 8-plex was chosen for protein quantification, and unique peptides/proteins with a global FDR of < 1% were considered for further analysis.

### Bioinformatic analysis of differential abundance proteins (DAPs)

To identify DAPs from the protein data, we used the following criteria: number of unique peptides ≥2; fold change (FC) threshold for upregulation/downregulation = 1.5/0.67; and maximum allowed FC = 100. The website-based tools agriGO (http://bioinfo.cau.edu.cn/agriGO/analysis.php) and KOBAS (ver. 3.0; https://www.biostars.org/p/200126/) were used to analyze the DAPs identified for Gene Ontology (GO) enrichment and Kyoto Encyclopedia of Genes and Genomes (KEGG) pathway analysis. An online tool for generating Venn diagrams (http://bioinformatics.psb.ugent.be/webtools/Venn/) was used to visualize overlapping DAPs between groups (bh51 compared with bh50 and bh53 compared with bh50).

### RT-PCR assays

Total RNA from immature spike tissue was isolated using Trizol reagent (Invitrogen, Carlsbad, CA, USA**)** in accordance with the manufacturer’s instructions. Approximately 2 μg of DNA-free total RNA and a PrimeScript 1st Strand cDNA synthesis kit (TaKaRa Bio, Inc., Dalian, China) were used for first-strand cDNA synthesis. Quantitative real-time PCR reactions were performed on a Mastercycler ep realplex real-time PCR system (Eppendorf, Hamburg, Germany) with SYBR Premix Ex Taq (TaKaRa Bio, Inc.). The cDNA was amplified using specific primers (Additional file 1: Table S1). Primer pairs were designed for qRT-PCR using the local AlleleID software (ver. 6.0; http://www.softpedia.com/get/Science-CAD/AlleleID.shtml). The expression levels of the corresponding genes are presented relative to corresponding control samples under the indicated conditions, with normalization against the Tubulin internal control gene (F: GACGCATGTCCATGAAGGAG; R: CCAATGCAAGAAAGCCTTGC). Relative gene expression levels were calculated using the 2^–△△CT^ method [22]. Correlation coefficients and histograms were calculated and plotted using R software for statistical computing (ver. R-3.0.0; http://www.r-project.org).

## Results

### The developmental characteristics of RSs and FRSs during the spikelet initiation phase

To determine the developmental characteristics of spikes in the NILs, seedling morphology and spike architecture were examined. The bh51 and bh53 lines had classic SS traits, and both had more spikelets and grains per spike than NS phenotype (bh50, wild-type) plants (Fig. 1) [2]. Immature spikes from the main tiller of the stem were collected at the seedling stage for further examination by scanning electron microscopy. The results showed that lateral meristems emerged simultaneously between the glume and lemma, indicating that this could be a key stage in the transformation of the spikelet meristem to a floret meristem. Tissues from all three NIL genotypes were harvested for 8-plex iTRAQ analysis.

### The principal component (PCA) and cluster analysis

**Fig. 2:**
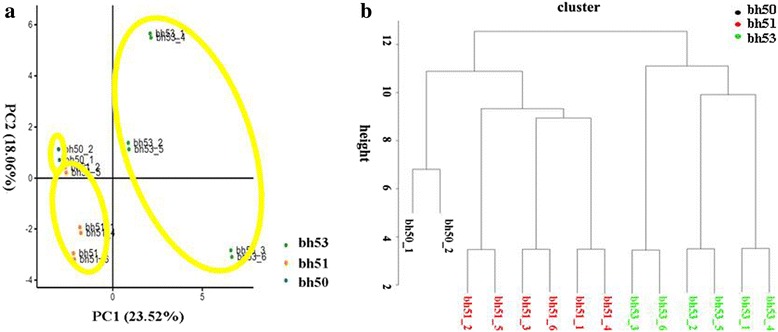
Principal component (PCA) and cluster analyses of differential abundance proteins (DAPs) using individual replicates. A: Principal component analysis (PCA) of NILs. Data are expressed as relative values: bh53 is shown in green; bh51 is shown in orange; and bh50 is shown in blue. B: The individual replicates were separated into three groups: bh51 is shown in red; bh53 is shown in green; and the two bh50 controls are shown in black

An iTRAQ-based shotgun quantitation approach was used to compare overall differences in the proteomes of the three experimental groups (bh50, bh51, and bh53). A total of 6249 proteins were identified with FDR < 1%, and 3834 changes in protein abundance were assessed (Additional file 2: Table S2). As a starting point for the DAP analysis, expression data were used to determine the global relationship between the different genotypes. PCA was performed using the individual replicates from each genotype. The results demonstrated that although the first two principal components could only explain 41.58% of the variation, the data points in the graph formed tight clusters, especially the controls (Fig. 2a). Additionally, we were able to separate the replicates from each genotype into three discrete groups (Fig. 2b), which is a further indication that the data are reliable.

### Expression patterns of DAPs

**Fig. 3:**
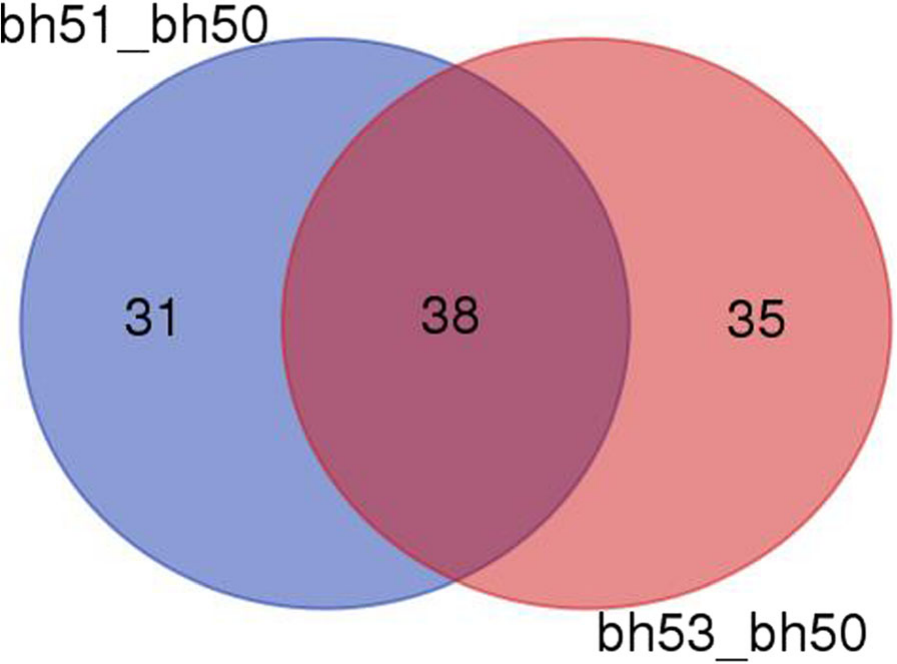
Venn diagram showing proteins co-expressed in FRSs and RSs compared with NSs

Applying the cut-off threshold of a 1.5-fold change for increased accumulation and a 0.67-fold change for decreased accumulation, together with the number of unique peptides ≥2, totals of 70 and 74 proteins showed differential accumulation in the bh51 versus bh50 and bh53 versus bh50 groups, respectively. Among these DAPs, 25 proteins were upregulated and 45 were downregulated in bh51 compared with wild type (bh50). In contrast, the expression of 27 proteins was increased and 47 decreased in bh53 compared with bh50 (Additional file 3: Table S3). When a Venn diagram was used to determine common DAPs among the three experimental groups, 27 proteins were unique to the bh51/bh50 group, 24 were unique to the bh53/bh50 group, and 38 were shared between the two groups (Fig. 3). Additionally, almost all the shared DAPs exhibited the same fold-change trends. For example, 97.37% of the proteins were either upregulated or downregulated when bh50 protein expression was compared with bh51 and bh53 expression, whereas only one protein (W5DNF1) changed the direction of its expression between the two experimental groups (Additional file 3: Table S3).

### Functional annotation and categories of DAPs

**Fig. 4:**
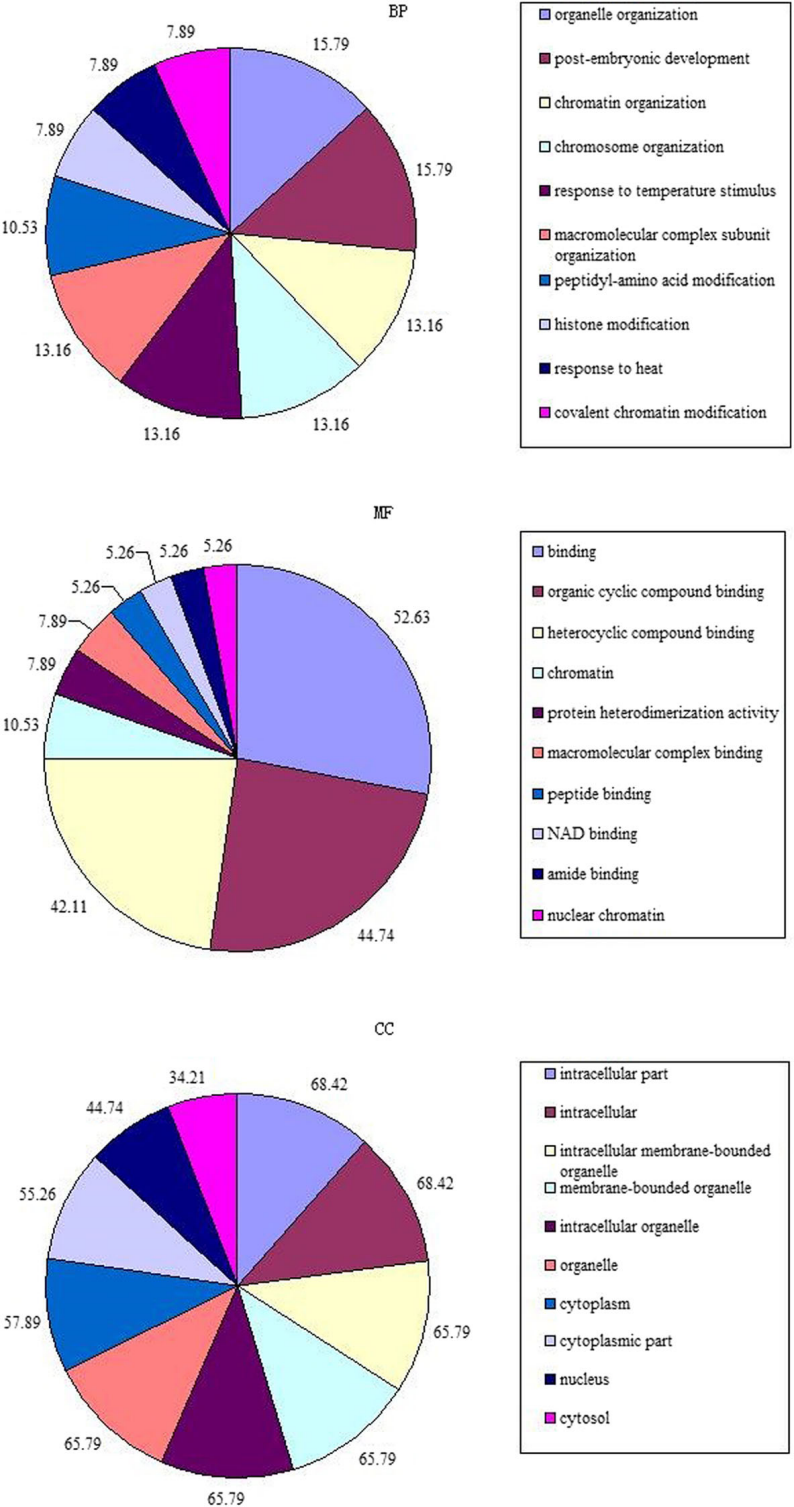
Gene Ontology and Kyoto Encyclopedia of Genes and Genomes analyses of the DAPs. Functional analysis of the proteins included three categories: biological processes (BP), cell components (CCs), and molecular function (MF). Counts for each category represent the functional descriptions in the database associated with the query proteins. Descriptions with *p* < 0.05 are statistically significant

The proteins that overlapped in the two experimental groups (38 DAPs) were analyzed using bioinformatics to understand the molecular pathways and processes involved. A total of 12 proteins were annotated in the biological process (BP) category, and 20 terms were significantly enriched. In contrast, 26 proteins were annotated in the cell component (CC) category, and 20 terms were significantly enriched. A total of 20 proteins were annotated in the molecular function (MF) category, including 10 significantly enriched terms (Fig. 4). In the BP analysis, “post-embryonic development” and “organelle organization” accounted for the largest proportion of DAPs (adjusted *p*-value = 4.63E^− 02^), followed by biological processes that included “chromosome organization”, “peptidyl-amino acid modification”, and “histone modification” (Additional file 4: Table S4). The CC analysis demonstrated that most of the annotated proteins were intracellular. Binding activity was the dominant MF among the GO assignments.

### KEGG analysis provides more information on spikelet development

The results of the KEGG analysis identified seven significant pathways, among which “metabolic pathways” was the most significant (adjusted *p*-value = 4.15E^− 03^) (Additional file 4: Table S4). Proteins of particular interest included O49485 (W5ASV5) and P25858 (W5HB91) because these proteins are involved in “metabolic pathways” and “post-embryonic development” according to the BP analysis (*p* = 3.26E^− 02^) (Additional file 4: Table S4; Table 1). The results of GO and KEGG analysis indicated that the changed expression model of enriched proteins involved in developmental process and metabolic compounds will contribute to the differentiation of lateral meristem.

**Table 1:** The further annotations of 38 DAPs in *triticum* and their correspondingly homeologous proteins in *Arabidopsis*

Ensempled ID	Uniprot ID	bh51_bh50	bh53_bh50	*Arabidopsis thaliana*	Annotation	Background
Traes_1DS_8BF71428D	W5 AM41	0.200	0.151	A0A178UPQ7	replication protein A 70 kDa DNA-binding subunit C-like	*Aegilops tauschii*
Traes_1BS_C1367433E	A0A096UKP8	0.276	0.211	Q9S7C0	Heat shock 70 kDa protein 4 L	*Triticum urartu*
Traes_3AS_3CB8A9C01	W5CJQ7	0.319	0.125	A0A178UK63	glutamate decarboxylase-like	*Aegilops tauschii*
TRIAE_CS42_5BL_TGACv1_404429_AA1299600	W5F969	0.358	0.393	P26569	histone H1	*Triticum aestivum*
TRIAE_CS42_4AS_TGACv1_306373_AA1007220	P12463	0.404	0.589	P83755	photosystem II protein D1	*Triticum aestivum*
Traes_2DS_5AA2EEDB7	W5C595	0.408	0.234	Q05431	L-ascorbate peroxidase 2, cytosolic	*Aegilops tauschii*
Traes_1DL_FDB539EBE	W5AJW7	0.439	0.376	Q6TBX7	carotene epsilon-monooxygenase, chloroplastic	*Aegilops tauschii*
Traes_2AL_608FCBC83	W5ASV5	0.476	0.531	O49485	D-3-phosphoglycerate dehydrogenase, chloroplastic	*Triticum urartu*
TRIAE_CS42_1DS_TGACv1_080841_AA0254500	W5ALP4	0.530	0.577	O22898	long chain acyl-CoA synthetase 1	*Aegilops tauschii*
Traes_7AL_D93FC054C	W5HB91	0.551	0.319	P25858	glyceraldehyde-3-phosphate dehydrogenase 1,cytosolic	*Aegilops tauschii*
Traes_4DL_852DF544C	W5EJA0	0.568	0.529	Q94A40	coatomer subunit alpha-3-like	*Aegilops tauschii*
Traes_7BS_3FE10DB62	W5HTI2	0.573	0.640	Q9FJH6	ABC transporter F family member 1-like	*Aegilops tauschii*
Traes_5BL_5A9F5F455	W5FBW2	0.574	0.567	A0A178VSW7	long chain acyl-CoA synthetase 8	*Aegilops tauschii*
TRIAE_CS42_3B_TGACv1_223285_AA0779670	A0A077RWA8	0.591	0.463	BLH8	BEL1-like homeodomain protein 8	*Aegilops tauschii*
TRIAE_CS42_4AL_TGACv1_288314_AA0944510	W5DNF1	0.592	1.751	Q9M0F5	Acid phosphatase 1	*Triticum urartu*
Traes_2BS_F83DB6517	W5BQ81	0.593	0.458	Q9MAB3	probable nucleolar protein 5–2	*Aegilops tauschii*
Traes_3DL_0F45E971E	W5D7U7	0.603	0.622	F4INY4	DExH-box ATP-dependent RNA helicase DExH6-like	*Aegilops tauschii*
TRIAE_CS42_4AS_TGACv1_307610_AA1021920	W5DWN6	0.611	0.579	Q94AH8	alpha,alpha-trehalose-phosphate synthase [UDP-forming] 6-like	*Aegilops tauschii*
Traes_6AS_ADCD6AB01	W5GFL5	0.616	0.612	Q8VZH2	aminopeptidase M1-A	*Aegilops tauschii*
Traes_1BL_4D2CB33FC	W5A1T0	0.616	0.635	Q84TF0	aldo-keto reductase family 4 member C10-like	*Aegilops tauschii*
Traes_1BS_E759ABAC6	W5ABV2	0.618	0.533	Q9SR03	Ankyrin-1	*Aegilops tauschii*
Traes_2AL_396E0F5A3	W5AR89	0.641	0.666	A0A178VJM6	60S ribosomal protein L23a	*Aegilops tauschii*
Traes_5BL_1923931ED	W5F841	0.651	0.570	Q94AK8	mRNA turnover protein 4 homolog	*Aegilops tauschii*
TRIAE_CS42_1BL_TGACv1_030547_AA0093660	W5A3B9	0.664	0.582	Q9ZVD5	protein argonaute 4B-like	*Aegilops tauschii*
Traes_1BL_F63A1C348	W5A7F0	1.580	1.560	Q9LSQ5	NAD(P)H dehydrogenase (quinone) FQR1-like	*Aegilops tauschii*
Traes_6DL_0CB0CD181	W5GU54	1.598	2.075	Q9C566	peptidyl-prolyl cis-trans isomerase CYP40-like isoform X1	*Aegilops tauschii*
TRIAE_CS42_5BL_TGACv1_404160_AA1287480	W5FH53	1.652	1.820	Q84L30	putative DNA repair protein RAD23	*Aegilops tauschii*
TRIAE_CS42_5AL_TGACv1_377373_AA1246230	A0A1D5YPJ3	1.676	1.517	Q9ATB4	transcription factor activity	*Aegilops tauschii*
TRIAE_CS42_3AL_TGACv1_193884_AA0621700	A0A077S7Z9	1.690	1.619	Q9LHG9	ribosome-nascent	*Aegilops tauschii*
TRIAE_CS42_5DS_TGACv1_457027_AA1481190	W5G4W4	1.727	1.607	Q8LD42	spore wall protein 2-like isoform X1	*Aegilops tauschii*
TRIAE_CS42_4DL_TGACv1_343955_AA1141980	A0A096URG5	1.803	1.744	A0A178UL04	DNA binding	*Aegilops tauschii*
Traes_4BL_8DFEB9631	W5E5A9	1.888	1.645	P59226	Core component of nucleosome	*Aegilops tauschii*
TRIAE_CS42_4AS_TGACv1_306997_AA1016020	W5DZ32	1.897	1.672	Q9C500	Plays a role with HSP70–1	*Aegilops tauschii*
TRIAE_CS42_2AL_TGACv1_095539_AA0311970	W5AQ98	1.935	2.095	Q96520	Removal of H_2_O_2_	*Aegilops tauschii*
TRIAE_CS42_2BS_TGACv1_147513_AA0484110	W5BLU3	2.267	1.930	Q84W92	histone methylation	*Aegilops tauschii*
TRIAE_CS42_1AL_TGACv1_001923_AA0036820	W4ZPI7	2.274	1.851	A0A178V1F6	endopeptidase	*Aegilops tauschii*
Traes_6DL_2807D89841	W5GUY7	2.323	1.992	Q9LQQ4	histone H2B.2-like	*Aegilops tauschii*
Traes_4BL_58958C7B9	W5E3M3	2.388	1.806	Q8H0V1	CDK5RAP1-like protein	*Aegilops tauschii*

### Temporal and spatial expression of candidate genes

**Fig. 5:**
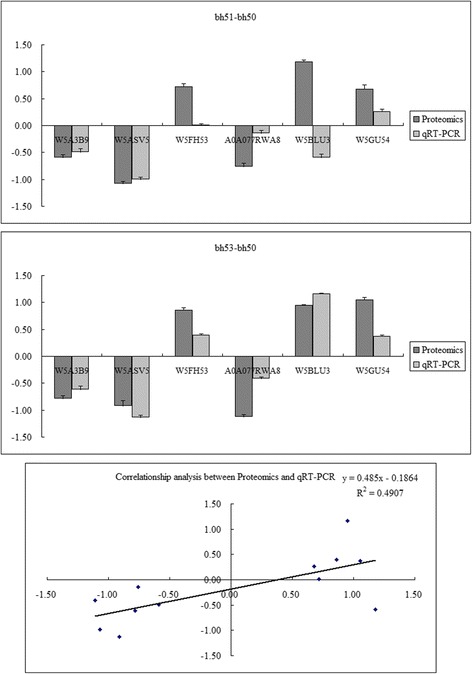
The spatiotemporal expression characteristics and correlation between the proteomic and quantitative real-time polymerase chain reaction data from the DAPs among the different groups

To look for correlations between mRNA levels and protein abundance, transcriptional analysis of six proteins was performed using qRT-PCR (Additional file 1: Table S1). Transcript levels of the genes and abundance of the corresponding proteins displayed similar trends for W5A3B9 (protein argonaute 4B-like), A0A077RWA8 (BEL1-like homeodomain protein 9), W5GU54 (peptidyl-prolyl cis-trans isomerase), W5ASV5 (D-3-phosphoglycerate dehydrogenase), and W5FH53 (putative DNA repair protein RAD23). The only exception was W5BLU3 (involved in histone methylation) for the bh51–bh50 group comparison. In general, W5A3B9, A0A077RWA8, and W5ASV5 were downregulated, whereas W5GU54 and W5FH53 were upregulated. Individually, the expression and abundance of W5BLU3 only increased in the bh53–bh50 group comparison, whereas there was a discrepancy between the proteomic and transcript data in the bh51–bh50 group comparison (Fig. 5). Quantitative analysis demonstrated that transcript levels of W5ASV5 measured by qRT-PCR in the bh51–bh50 and bh53–bh50 group comparisons changed 0.505- and 0.456-fold, respectively, whereas the corresponding protein levels were altered 0.476- and 0.531-fold. The linear regression equation (R^2^ = 0.4907) and correlation coefficient (0.7005) are also shown in Fig. 5.

## Discussion

A major challenge for wheat breeders is to find stable and sustainable ways of increasing yield potential, with a particular focus on the genetic improvement of panicle traits. Although extensive research has investigated supernumerary and branched spikelet phenotypes using various strategies, and vital genes such as *WFZP* have been identified [2, 7, 23], little is known about the genetic pathways leading to spikelet formation in tetraploid wheat. Here, a proteomic approach was used to investigate the molecular mechanisms of branched spike development.

**Fig. 6:**
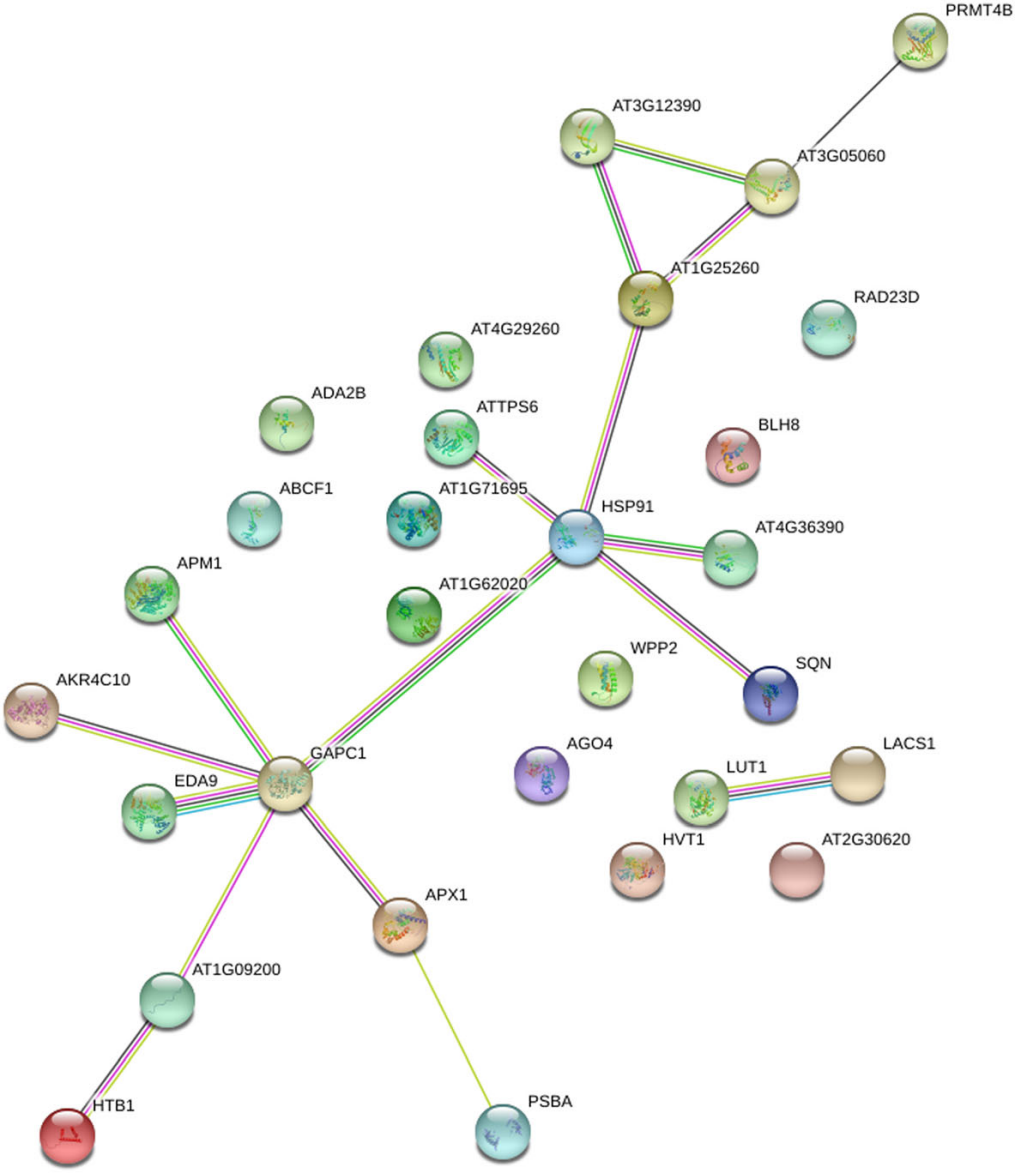
Putative molecular regulatory networks from the NILs

A total of 104 DAPs were identified by comparing the NILs bh50 with bh51 and bh53 using the FC criteria ≥1.5 or ≤ 0.67 (Fig. 2) and the number of unique peptides ≥2. Among these, only 38 DAPs were common to both the bh51 and the bh53 group comparisons with bh50, and these were used to construct a network model for lateral meristem development. Further GO and KEGG pathway analyses provided vital biological messages for putative molecular network construction that could affect the formation and development of young spikelet tissues (Fig. 6). As a result, many genes related to these BPs were annotated as potential targets for future experimental analysis. The functional description of “post-embryonic development” (0009791) was associated with six proteins, namely, W5C595, W5ASV5, W5HB91, W5GU54, W5DZ32, and W5BLU3. Additionally, W5BLU3 was involved in “histone modification” (GO: 0016570) or “protein methylation” (GO: 0016571) pathways. The significance and proportion of enriched BPs suggested that “organelle organization” and “post-embryonic development” may be key processes in controlling spikelet differentiation. Therefore, some of the key proteins in the network associated with these biological processes were investigated further.

### Putative GO and KEGG analysis networks

To examine some of the regulatory networks more closely, DAPs of particular interest were submitted to the STRING database for interactive pathway analysis based on data from *Arabidopsis* (Additional file 5: Table S5) [24]. The pathways/functions include regulation of transcription factor activity (BLH8), regulation of transcription (ADA2B, AGO4), glycolysis (GAPC1), removal of reactive oxygen species (APX1), heat shock protein-related (Hsp90.1, WPP2) and probable histone-arginine methyltransferase (PRMT4B). Because the number of DAPs was limited, independent spots, such as protein folding (CYP40) and even serine biosynthesis (PGDH1), are not shown in the network in Fig. 6. This information provides insight into the mechanism of lateral meristem growth, and possible interactions between these proteins could be tested by yeast two-hybrid experiments in the future.

*Wfzp* is a member of a gene family characterized by two AP2 domains. Other members include APETALA 2 and proteins that participate in inflorescence meristem development [25, 26]. Unlike ERF-type proteins, these AP2-type transcription factors usually bind to the promoters of genes containing the consensus motif: 5’-gCAC(A/N)N(A/T)TcCC(a/g)ANG(c/t)-3′ [27]. Additionally, all of them contain an miR172 target site, including *TARGET OF EAT1* (*TOE1*), *TOE2*, *TOE3*, *SCHLAFMUTZE* (*SMZ*), and *SCHNARCHZAPFEN* (*SNZ*) in *Arabidopsis* [28, 29]. Many of these regulatory pathways have been investigated in model plants. Zhu (2011) proposed a conserved regulatory network with key roles for miR156 and miR172 in triggering changes in the developmental phase and in flower development in *Arabidopsis* and monocotyledons [30]. When over-expressed in plants, the 21-nucleotide non-coding microRNA miR172 was able to downregulate AP2 target genes, including *IDS1* [31], *SID1* [32], and *SNB* [33]. In this study, we also identified downregulated proteins (e.g., W5A3B9) that bound to miR172 and were involved in targeting AP2 mRNAs and mediating the cleavage of miRNA targets. Additionally, A0A077RWA8, which acts synergistically with BLH8 in *Arabidopsis*, is shown in the network to interact with AP1-like proteins, including LFY [34] and AGL8 [35]. The fact that our results are consistent with previous findings confirm their reliability. We further investigated some of the key proteins in the network that are associated with major biological processes to examine any links between their functions and inflorescence differentiation, although the precise nature of any interaction between these proteins has yet to be established.

### Histone modification and post-transcriptional gene regulation

The W5A3B9 protein and its *Arabidopsis* homolog ARGONAUTE 4 (AGO4) have important roles in RNA-mediated silencing systems, including post-transcriptional gene silencing in plants [36]. Additionally, 24-nt miRNAs, including miR172, are recruited by AGO4 to induce methylation and silence target genes [30, 37, 38]. The expression level of AGO4 protein decreased in both of our group comparisons, and similar results were observed in our qRT-PCR experiments (Fig. 5). Although the expression level of miR172 in the NILs is unclear, we predict that the target AP2-like genes could be upregulated. Previous results demonstrated that growth inhibition in axillary meristems was affected by single-nucleotide polymorphism mutations in the ORF of *WFZP*. Therefore, mutations in *FZP* may stimulate ectopic development at the spikelet meristem and influence organ identity at the floral meristem [4, 6, 7].

Histone methyltransferases are histone-modifying enzymes (e.g., histone-lysine N-methyltransferases and histone-arginine N-methyltransferases) that catalyze the transfer of methyl groups to histone lysine and arginine residues [39]. Methylated histones can either repress or activate transcription [40]. W5BLU3 is orthologous to *Arabidopsis* PRMT4B and may be recruited to promoters upon histone H3 methylation, activating transcription via chromatin remodeling [41]. In our study, the expression of this protein was increased in both group comparisons and in the qRT-PCR results comparing bh53 with bh50, which suggests that it may activate the expression of downstream genes. Additionally, FRIGIDA-like protein 4a (FRL4A) in *Arabidopsis* not only regulates flower development but also participates in cell proliferation [42]. In rice, orthologs of *PRMT5* appear to be key mitotic regulators and are essential for the accurate pre-mRNA splicing that regulates flowering in plants [43].

In *Arabidopsis*, the transcriptional adapter ADA2b (a homolog of A0A1D5YPJ3) is required to stimulate acetyltransferase activity of *GCN5* on free histones or nucleosomes by opening up the promoter region [44]. It may also mediate auxin and cytokinin signaling to control cell proliferation and play a role in repressing freezing tolerance pathways at warmer temperatures [45]. Because the A0A1D5YPJ3 protein was upregulated in both our bh51 and bh53 proteomic group comparisons, this gene may promote transcriptional activity in lateral meristem development.

### DAPs in the ubiquitination pathway could affect protein degradation

The ubiquitin pathway plays important roles in regulating cell cycle progression via 26S-proteasome-mediated protein degradation and involves two different E3 ligase complexes called SCF (skp-cullin-F-box protein) and APC/C (anaphase-promoting complex). Many studies suggest that this pathway is also involved in regulating spike morphogenesis [46]. Ikeda et al. (2007) reported that *Aberrant Panicle Organization 1* (Os*APO1*) encodes an F-box protein that regulates spikelet and floral identity and determinacy in rice and is also an ortholog of *Unusual Floral Organ* (*UFO*) in *Arabidopsis* [47]. Additionally, the *Arabidopsis* AtDA1 protein can act as a ubiquitin receptor and regulate cell proliferation, producing larger floral organs [48]. Therefore, this pathway may also include other proteins relevant for our study. The ubiquitin receptor *RAD23d* associates with the 26S proteasome docking subunit RPN10 to facilitate the recognition of ubiquitinated substrates for ubiquitin/26S proteasome-mediated proteolysis [49]. RPN10 plays a role in balancing cell expansion with cell proliferation rates during shoot development [50]. The expression levels of RPN10 in both the proteomic and qRT-PCR results were elevated, which may indicate that protein degradation is accelerated. Therefore, the function and network associated with the *RAD23d* receptor gene should be investigated more thoroughly in future studies.

### Transcription factors involved in shoot development

BEL1-like homeodomain protein 8 (BLH8) is homologous to A0A077RWA8 and functions as a transcription factor maintaining stem cell fate in the shoot apical meristem, specifying floral primordia, and establishing early internode patterning events during inflorescence development [51]. It also represses the transcription of AGAMOUS (AG) in floral and inflorescence meristems [52]. Eleven BLH8 regulation network interactors were identified using the STRING database annotations (http://string-db.org/cgi/input.pl?UserId=oWlcBNoivY9Y&sessionId=8Pmofu57T0vk&input_page_show_search=on). These included AP1 (A-class MADS box gene) and a member of the KNOTTED class of homeodomain proteins (KNAT1, KNAT2, KNAT5, and KNAT6) encoded by the *STM* gene, which has a previously demonstrated role controlling development of the inflorescence meristem [53]. Additionally, a close correlation was observed between the proteomic studies of A0A077RWA8, which was downregulated approximately 2-fold in bh51 compared with that in bh50 (0.591) and in bh53 compared with that in bh50 (0.463). Because A0A077RWA8 represses AG (a C-class MADS box protein), it may play a role in floral meristem development by an unknown molecular mechanism.

### DAPs involved in carbohydrate and nitrogen metabolism

The *Arabidopsis* homolog of W5ASV5, D-3-phosphoglycerate dehydrogenase 1 (PGDH1), is required for the phosphorylated pathway of serine biosynthesis and for mature pollen development [54]. Phosphorylation of histone Ser residues is important for regulating cell division and development [55]. The proteomic and mRNA results from both of our group comparisons showed an approximately 2-fold decrease in the expression of W5ASV5. Because this enzyme is involved in an energy release process in glycolysis, the protein may act as a negative regulator in lateral meristem developmental processes. We will investigate this gene further in our next study.

The *Arabidopsis* homolog of W5HB91, glyceraldehyde-3-phosphate dehydrogenase (GAPC1), is a key enzyme in glycolysis and plays essential roles in the maintenance of cellular ATP levels and carbohydrate metabolism [56]. It is also involved in fruit and seed development because the *gapc-1* null mutant has aborted/empty embryonic sacs in its basal and apical siliques. The relationship between the RS and FRS phenotypes and decreased enzyme activity merits further study.

### Upregulated HSPs may be involved in developmental processes

Peptidyl-prolyl cis-trans isomerase (CYP40), which is orthologous to W5GU54, catalyzes the cis-trans isomerization of proline imidic peptide bonds in oligopeptides and is involved in promoting the juvenile phase of vegetative development, regulating the position of floral buds, floral morphogenesis, and the expression of HSPs [57]. In our study, the proteomic and qRT-PCR expression patterns for W5GU54 were consistent, demonstrating an upregulation in both the RS and FRS genotypes. Another vital protein, W5DZ32, which is homologous to WPP2 in *Arabidopsis*, was developmentally associated with the nuclear envelope and promoting cell division [58]. WPP2 could act together with HSP70–1 to facilitate WIT1 targeting at the nuclear envelope [59]. The upregulated expression from the proteomic assays suggests that these genes may promote a transition from vegetative to reproductive development.

## Conclusions

The SS traits in tetraploid wheat are crucial for improving grain number per spike, although the practical increasing potential under field environments still needs to be further examined. Using the iTRAQ-based shotgun quantitation approach, a total of 6249 proteins were identified with less than 1% FDR, and 3834 protein abundance perturbations were confidently assessed. Then, 38 co-expressed DAPs from 104 DAPs with < 1% false discovery rate (FDR) and a 1.5-fold change (> 1.50 or < 0.67) were selected for further bioinformatics analysis and putative network construction. We predicted that DAPs involved in “Post-embryonic development” and “Metabolic pathways” processes could partly account for the genomic, physical and biochemical changes in FRS and RS. We suspected that the change in DAP expression was predominantly functionally related to histone modification and regulation, ubiquitin-mediated protein degradation, transcription factors and carbohydrate and nitrogen metabolism. The results of qRT-PCR are partially consistent with these findings in proteomics. However, further experimental work is needed to confirm our prediction. This work will establish a foundation for elucidating the molecular mechanism underlying SS traits in tetraploid wheat, in addition to *WFZP*.

## Additional files

Additional file 1: **Table S1**. The information of qRT-PCR primers designed by AlleleID software. (XLS 24 kb)

Additional file 2: **Table S2**. Expression abundances of 3834 proteins among three experimental groups. (XLS 1132 kb)

Additional file 3: **Table S3**. The expressional models of 104 DAPs in bh51_bh50 and bh53_bh50. (XLS 29 kb)

Additional file 4: **Table S4**. Results of GO and KEGG analysis of 38 common expressed DAPs. (XLS 38 kb)

Additional file 5: **Table S5**. The networks of 38 DAPs by searching String database. (XLS 30 kb)

## Abbreviations

2D-LC: two-dimensional liquid chromatography
DAPs: Differential abundance proteins
FC: Fold change
FDR: False discovery rate
FRS: four-rowed spikelete
iTRAQ: isobaric tags for relative and absolute quantification
MS/MS: tandem mass spectrometry
NILs: Near isogenic lines
NS: Normal spikelete
PCA: Principal component analysis
qRT-PCR: quantitative real-time PCR
RPLC: Reversed-phase liquid chromatography
RS: Ramified spikelete
